# CT scan exposure in Spanish children and young adults by socioeconomic status: Cross-sectional analysis of cohort data

**DOI:** 10.1371/journal.pone.0196449

**Published:** 2018-05-03

**Authors:** Magda Bosch de Basea, Ana Espinosa, Mariona Gil, Jordi Figuerola, Marina Pardina, José Vilar, Elisabeth Cardis

**Affiliations:** 1 Radiation epidemiology, Barcelona Institute for Global Health (ISGlobal), Barcelona, Spain; 2 Universitat Pompeu Fabra (UPF), Barcelona, Spain; 3 CIBER Epidemiología y Salud Pública (CIBERESP), Madrid, Spain; 4 Hospital Universitari Arnau de Vilanova, Lleida, Spain; 5 Hospital Universitario Doctor Peset, Valencia, Spain; CUNY, UNITED STATES

## Abstract

Recent publications reported that children in disadvantaged areas undergo more CT scanning than others. The present study is aimed to assess the potential differences in CT imaging by socioeconomic status (SES) in Spanish young scanned subjects and if such differences vary with different indicators or different time point SES measurements. The associations between CT scanning and SES, and between the CT scan rate per patient and SES were investigated in the Spanish EPI-CT subcohort. Various SES indicators were studied to determine whether particular SES dimensions were more closely related to the probability of undergoing one or multiple CTs. Comparisons were made with indices based on 2001 and 2011 censuses. We found evidence of socio-economic variation among young people, mainly related to autonomous communities of residence. A slightly higher rate of scans per patient of multiple body parts in the less affluent categories was observed, possibly reflecting a higher rate of accidents and violence in these groups. The number of CT scans per patient was higher both in the most affluent and the most deprived categories and somewhat lower in the intermediate groups. This relation varied with the SES indicator used, with lower CT scans per patients in categories of high unemployment and temporary work, but not depending on categories of unskilled work or illiteracy. The relationship between these indicators and number of CTs in 2011 was different than that seen with the 2001 census, with the number of CTs increasing with higher unemployment. Overall we observed some differences in the SES distribution of scanned patients by Autonomous Community in Spain. There was, however, no major differences in the frequency of CT scans per patient by SES overall, based on the 2001 census. The use of different indicators and of SES data collected at different time points led to different relations between SES and frequency of CT scans, outlining the difficulty of adequately capturing the social and economic dimensions which may affect health and health service utilisation.

## Background

Almost 4 million computed tomography (CT) scans are performed annually in Spain [1], allowing for non-invasive detailed imagery of human anatomical inner structures. Despite its clinical advantages, the low to moderate doses of ionising radiation imparted in CT scanning have been associated with an increased risk of brain tumours and leukaemia in children and young adults [2–5].

Four recent papers have reported higher radiation doses and CT scan use in children living in disadvantaged areas [6–9]. In all of them, this finding was hypothetically attributed to a higher disease and injury rate compared to more affluent areas. The influence of the socioeconomic status (SES), the complex phenomenon that embodies economic, political, occupational and cultural differences among people, in the variation of disease burden among adults is widely accepted by the scientific community [10], but less evident for children, specially at early ages [11]. While little is known about brain tumours, higher incidence of childhood leukaemia (the most common type of childhood cancer) has been historically related with affluent communities in occidental societies [12]. Recent studies have challenged this long-held view associating higher childhood leukaemia rates with less affluent individuals [13]. A systematic review concluded that the results of these studies varied by the SES measures utilised [14]. Higher disease rates where associated with lower SES levels when individual–level measures of family income, mother’s education and father’s education were used. Occupational class, however, whether measured at the ecological or individual level, was positively associated with childhood leukaemia [14]. Different SES indicators could be capturing diverse risk factors, potentially explaining some of the observed differences in between studies.

Further, in most studies SES is studied as a static measure of the relative position of an individual within a hierarchical social structure, including a single-time-point SES measurement due to data availability and logistics. This may fail to realistically reflect the socioeconomic situation of a specific population in longitudinal studies, particularly after marked changes in the economy.

Universal health care coverage is intended to reduce social inequalities in health and burden of disease [15] but SES is still a strong predictor of differential use of health care services [16]. In Spain, though an overall increase in use of universal health care services has been observed in the last two decades, the use of specialised services is less frequent among those in the lower SES categories [16]. Nevertheless, lower SES individuals are more likely to use emergency services when compared to more affluent social classes [16]. Official Spanish indicators of CT scan distribution by educational level and socioeconomic status are available [1] though not for the 0–20 years of age. The comprehension of a possible differential use of CT imaging by socioeconomic strata in countries with universal health care may help to identify potential disparities in the delivery and use of health care services and in health risk perception, and contribute to assess the possibility that SES may confound the association between CT scan exposure and childhood diseases.

The objective of the present study was to determine whether there are differences in rates of CT imaging by SES in Spanish paediatric and young adult patients. The secondary aim was to study whether and how these differences may vary with the use of different indicators of social deprivation, as well as the implications of using SES indicators measured at different times.

## Methods

The Ethics Committee of the International Agency for Research on Cancer approved the study protocol (IARC IEC 12–35). The protocol has also been approved by all appropriate hospital ethics committees and appropriate authorities in Spain, prior to commencing the epidemiological study.

### Study population

EPI-CT, a multinational study to evaluate the relation between radiation dose from CT scans in young population and cancer risk, is currently underway [17]. The present analysis is based on the Spanish EPI-CT subcohort and includes patients who received at least 1 CT scan when they were younger than 21 years old between 1991 and 2013, in twenty public or autonomically-subsidised hospitals (private hospitals with governmentally contracted services) of five Spanish autonomous communities (AC): Catalonia, Valencia Community, Murcia region, Navarre and Madrid Community. Hospitals from the Basque country were excluded because patient residence was not available. All participating hospitals belong to the Spanish National Public Healthcare System, which is the sole health care provider for 86.2% of the general population, ranging from 73.7% in the Community of Madrid to 95.6% in Navarre [18]. Among young people, the Public Health system is the exclusive healthcare provider for 83.9, 86.8 and 89.6% of patients in the 0–4, 5–14 and 15–24 years-old age groups, respectively [18].

### Radiological data source

Information on CT scans was obtained from the Radiological Information System (RIS) since its implementation in the hospitals (between 1991 and 2010) until December 2013. An adaptation of the categories defined by Mettler [19] was used to group the examination descriptions into six categories: head/neck, thorax, abdomen/pelvis, spine, extremities and “several parts” (a composite of several scan locations scanned in a single examination, e.g. head and thorax), plus the additional “unknown” category, when information on the anatomical area scanned was unavailable.

### Socioeconomic status

The residential address of each patient, as reported at his/her last CT scan or hospital visit (and the latest available address for 0.5% patients with reported address) was abstracted from the RIS in 2015, and geocoded using ARCGis (Esri, United States), Cartociudad (Instituto Geográfico Nacional, Ministerio de Fomento, Spain), ICC (Institut Cartogràfic i Geològic de Catalunya, Generalitat de Catalunya, Spain) and Google maps (Map data, Google, United States). For 24,604 addresses, the software did not provide any reliable location and addresses were manually geocoded. The geocoded addresses of the participants were linked to the Population and Housing Census to obtain the census tract to which they belonged. Each census tract includes, on average, 2500 people and it is characterised by a set of values for several social indicators and indexes compiled in the Atlas of Vulnerability [20] (Spanish Ministry of Development). The main socioeconomic index used in our analyses, the Urban Vulnerability Synthetic Index (UVSI), is calculated as the census-tract percentage of 5 socioeconomic indicators (proportion of unemployed population, youth unemployment, uneducated population (including illiterate and unschooled population), temporary employment and unskilled employment), each one of which is standardised to the average national level. The UVSI is based on 2001 census data and tract limits (unavailable for 2011) and ranges between 0 (less vulnerable) and 1 (more vulnerable). Additionally, each of the 5 individual indicators included in the UVSI were used to examine whether one SES dimension was more closely related to CT scan exposure. To assess the impact of using an up-to-date SES versus the traditionally used one-point SES-estimation, both 2001 and 2011 census data on the 5 SES indicators previously mentioned were obtained and assigned to those patients who had their last CT scan/hospital visit past 2006 (70.01% of all geocoded patients). The year 2006 was selected as the cut-off point because it marked the mid-point between the 2001 and 2011 censuses. Therefore for those subjects whose last CT scan/hospital visit happened past 2006 (and therefore, their residence address was updated in this last visit) we had complete certainty that their SES level is assigned using the latest socioeconomic indicators available.

### Statistical methods

In order to identify differences that could bias any relation between CT scans and SES, the homogeneity of the demographic characteristics between geocoded and non-geocoded individuals was tested using a Chi square test for independence. The association between SES and demographic characteristics of the study participants were studied using the Kruskal-Wallis and Fisher’s exact tests for continuous and categorical variables, respectively. Generalised Additive Models (linear and splines) were used to examine the relation between rate of CTs per patient and the different SES measures. The ratios of number of scans per patient in the different quintiles of SES to the number in the most affluent quintile, modelled as incidence rate ratio (IRR), were estimated using mixed effect negative binomial models including Autonomous Community (AC) of residence as a random effect, and sex and age at the time of the last CT scan because its inclusion supposed a change in the parameter estimate greater than 10%. A robust estimator of variance was used to account for the overdispersion within cluster-correlated data. The correlation between the SES summary index (UVSI) and each of the 5 indicators which constitute it (standardised to the average national value) was evaluated. For those patients who had their last CT scan past 2006, the relationship between CT scan rate per patient and SES was analysed using both 2011 and 2001 census-tract data, to assess the impact of updating the SES indicator after a major economic event, fitting mixed effect negative binomial regression models using a robust estimator of variance and a random effect component. Statistical association was set at a 0.05 significance level and a two-sided alternative hypothesis. Data analysis was performed using STATA 14.0 (StataCorp LP, Tx USA).

## Results

Over 79.7% (123,729 individuals) of the 155,309 children and young adults who received at least one CT scan between 1991 and 2013 in the participating hospitals and resided within the 5 AC had sufficient data to geocode their address to the census-tract level. Success of the geocoding process was independent of the number of scans per patient (p = 0.09) (S1 Table).

Within our population, the most prevalent scanned anatomical areas were the head/neck (60.6%), the thorax (14%.01) and the abdomen/pelvis (7.2%) and across the different age groups although with different relative frequencies (72.2%, 16.8% and 3.5% among age group <1year and 51.2%, 10.7% and 11.6% among age group 20 years) (data not shown). No differences were observed by gender. No information on CT scan indication was available.

### Socio-economic differences in CT scanning

**Fig 1:**
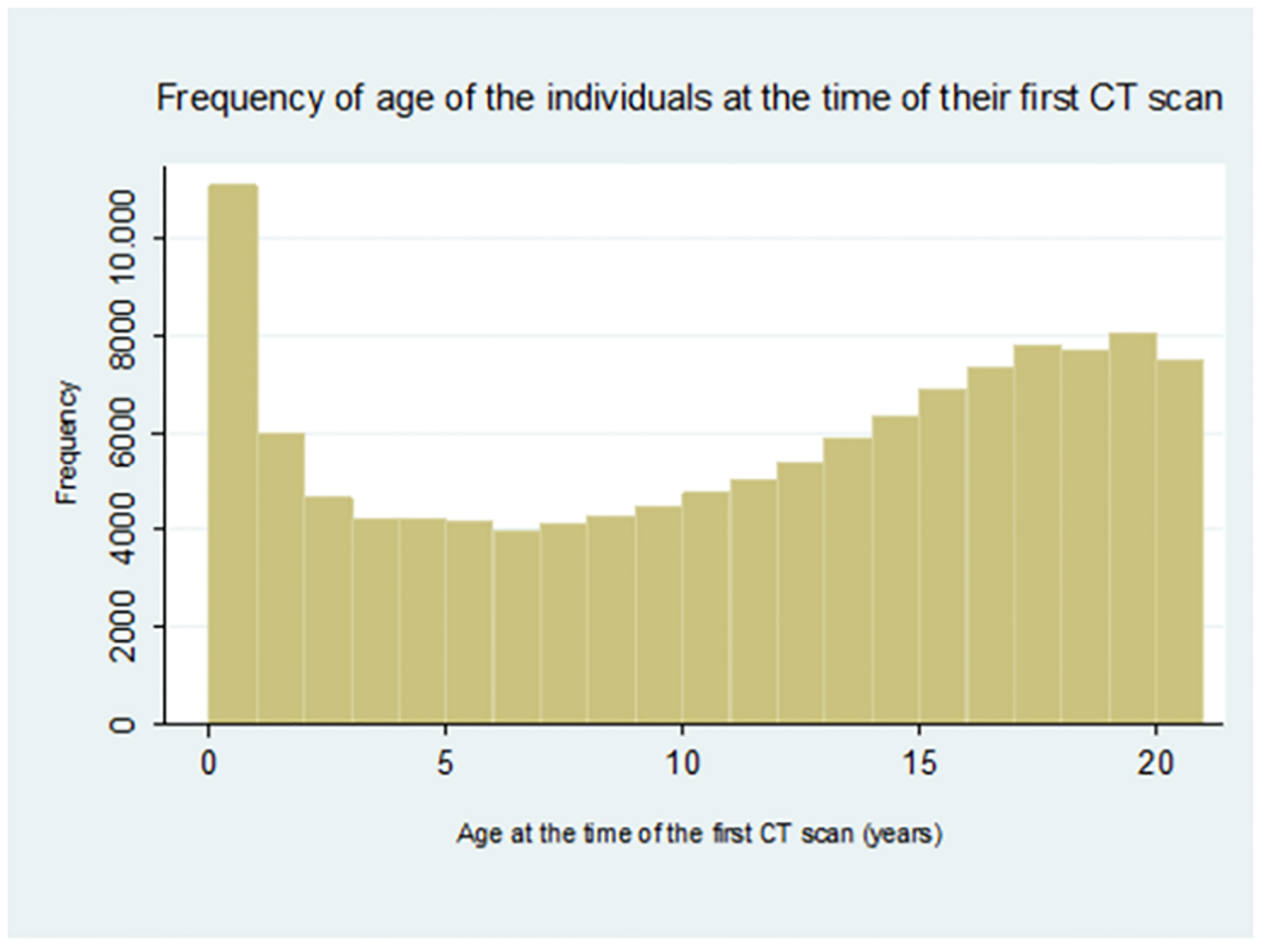
Frequency of age of the individuals at the time of their first CT scan.

A total of 205,541 CT scans were performed in 123,729 individuals. The distribution of age at the time of the first CT scan follows a bimodal distribution, with 8.1% of scanned patients below 1 year old and over 32.1% of patients being15 years or more (median = 12.2 years and 25^th^– 75^th^ percentile = 5.2–17.0 years) (Fig 1).

Characteristics of the patients are given in the Table 1 by UVSI quintiles. Patients in the 1^st^ and 2^nd^ quintiles (more advantaged groups) were slightly younger than in the lower quintiles. 56.3% of scanned patients were males, with a similar distribution across UVSI quintiles. The different population sizes and study periods in the participating ACs led to an unequal contribution of subjects to the study, with Catalonia providing, overall, almost half of the scanned patients (49.0%), followed by the Madrid (25.0%) and Valencia Communities (14.9%). The population distribution by SES quintiles varied substantially between ACs of residence; in Catalonia and Navarre privileged patients where overrepresented whereas in Murcia region, and Madrid and Valencia Communities a higher percentage of disadvantaged population was observed.

**Table 1:** Characteristics of the patients by quintiles of the Urban Vulnerability Synthetic Index—UVSI (2001 census data).

Patient characteristics	Total	1 (Less vulnerable)	UVSI			5 (More vulnerable)
			2	3	4	
	N = 123,729	N = 26,252	N = 25,363	N = 22,645	N = 25,520	N = 23,949
**Age at the time of the first scan—Median [25**^**th**^ **-75**^**th**^ **percentile]**	12.2 [5.2–17.0]	12.0 [5.4–16.9]	12.0 [5.1–16.9]	12.2 [5.2–17.0]	12.4 [5.2–17.1]	12.4 [5.0–17.2]
**Sex—N (%) patients**						
Males	69,681 (56.3)	14,923 (21.4)	14,209 (20.4)	12,626 (18.1)	14,307 (20.5)	13,616 (19.5)
Females	54,048 (43.7)	11,329 (21.0)	11,154 (20.6)	10,019 (18.5)	11,213 (20.7)	10,333 (19.1)
**Autonomous Community residence—N (%) patients**						
Catalonia	60,635 (49.0)	15,568 (25.7)	14,605 (24.1)	10,993 (18.1)	10,965 (18.1)	8,504 (14.0)
Madrid Community	30,884 (25.0)	5,743 (18.6)	4,574 (14.8)	5,160 (16.7)	7,487 (24.2)	7,920 (25.6)
Murcia region	4,618 (3.7)	195 (4.2)	349 (7.6)	626 (13.6)	789 (17.1)	2,659 (57.6)
Navarre	9,197 (7.4)	3,137 (34.1)	2,811 (30.6)	2,051 (22.3)	928 (10.1)	270 (2.9)
Valencia Community	18,395 (14.9)	1,609 (8.7)	3,024 (16.4)	3,815 (20.7)	5,351 (29.1)	4,596 (25.0)
**Body part scanned in first CT—N (%) patients**						
Head and neck	80,390 (65.0)	16,338 (20.3)	16,281 (20.3)	14,752 (18.4)	16,921 (21.0)	16,098 (20.0)
Thorax	12,975 (10.5)	2,879 (22.2)	2,677 (20.6)	2,293 (17.7)	2,673 (20.6)	2,453 (18.9)
Abdomen and pelvis	7,011 (5.7)	1,568 (22.4)	1,524 (21.7)	1,216 (17.3)	1,416 (20.2)	1,287 (18.4)
Spine	4,233 (3.4)	1,006 (23.8)	886 (20.9)	769 (18.2)	844 (19.9)	728 (17.2)
Extremities	6,745 (5.5)	1,362 (20.2)	1,299 (19.3)	1,152 (17.1)	1,539 (22.8)	1,393 (20.7)
Several parts	3,243 (2.6)	613 (18.9)	483 (14.9)	551 (17.0)	695 (21.4)	901 (27.8)
Unknown	9,122 (7.4)	2,485 (27.2)	2,213 (24.3)	1,912 (21.0)	1,431 (15.7)	1,081 (11.9)
**Number of CT scans—N (%) patients**						
1	89,709 (72.5)	19,088 (21.3)	18,410 (20.5)	16,552 (18.5)	18,493 (20.6)	17,166 (19.1)
2	29,940 (24.2)	6,209 (20.7)	6,098 (20.4)	5,343 (17.8)	6,234 (20.8)	6,056 (20.2)
3–10	3,053 (2.5)	699 (22.9)	641 (21.0)	555 (18.2)	603 (19.8)	555 (18.2)
11–20	905 (0.7)	227 (25.1)	181 (20.0)	172 (19.0)	170 (18.8)	155 (17.1)
> 20	122 (0.1)	29 (23.8)	33 (27.0)	23 (18.9)	20 (16.4)	17 (13.9)

65.0% of all first CT examinations were performed in the head and neck, followed by thorax (10.4%) and abdomen and pelvis (5.6%), and a similar distribution was observed across UVSI quintiles. Results were similar when considering all CT scans rather than the first CT (not shown). The scans involving several body locations were significantly more frequent in the two lower SES quintiles (21.4% and 27.8% respectively) whereas the highest percentage of examinations of unknown anatomical area was observed in the most affluent quintiles (Table 1).

During the study period (1991–2013) 72.5% of the patients received 1 CT scan, 24.2% received 2 and a very small fraction (3.3%) received 3 or more (Table 1). The proportion of subjects with 11 CTs or more was highest in the most affluent categories and decreased with decreasing level of SES. In general, the most affluent group tended to have more examinations than the other categories.

### Variation in number of CT scans per patient by SES

**Fig 2:**
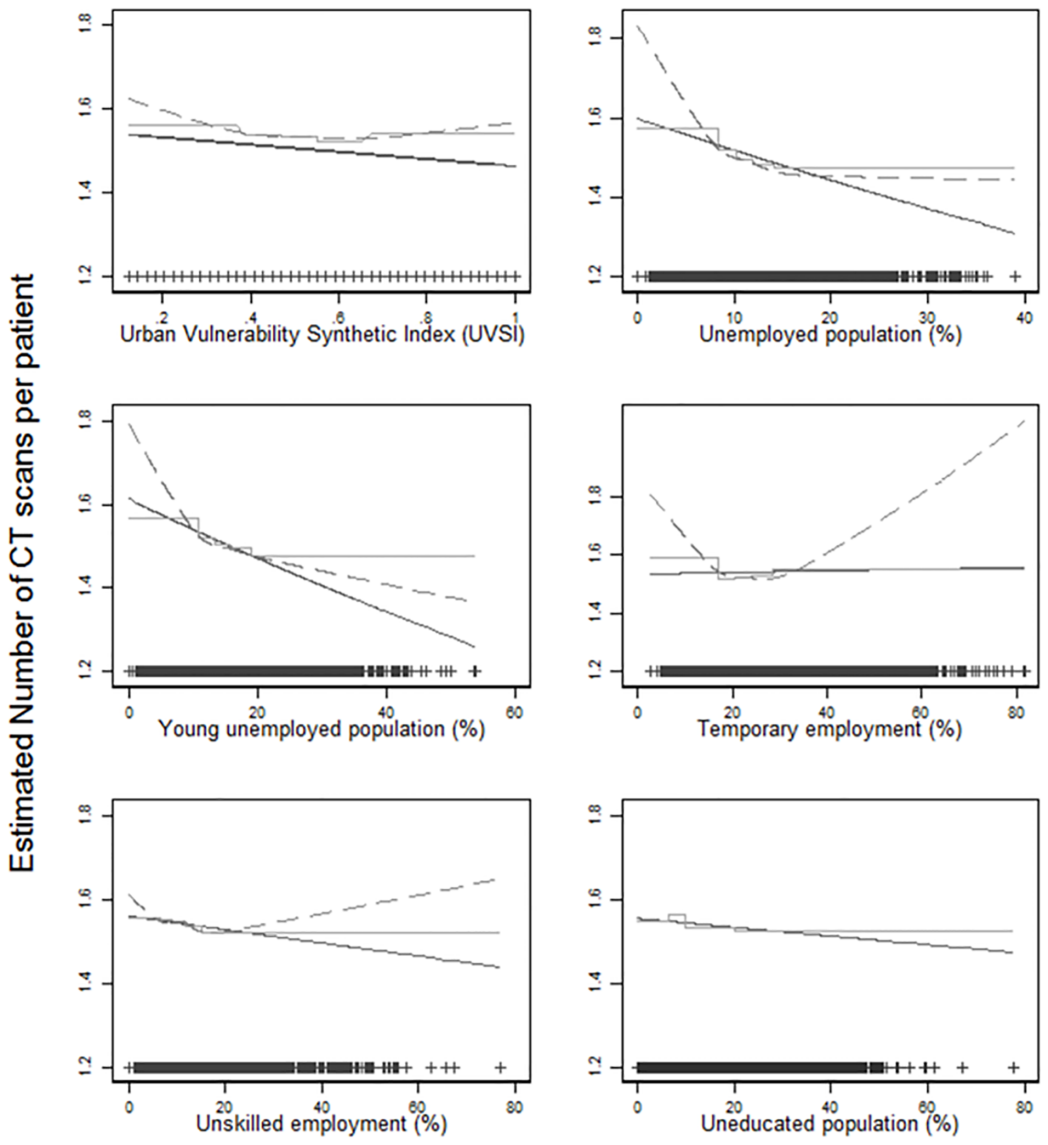
Estimated number of CT scans per patient as a function of the SES measured using the UVSI and its 5 constituent indicators. Results for continuous measure (solid line), categorical variable in quintiles (step function) and cubic spline model (dashed curve), adjusted for sex and age at the time of the last CT scan and including the autonomous community of residence as a random effect. Scatter points at the bottom are the observed values of the SES measure.

The relationship between the total number of CT scans per patient and UVSI was not linear (Fig 2), with the rate being highest in the 2 most privileged followed by the 2 least privileged quintiles and lowest in between. Cubic splines did not adequately describe this relationship, as shown with categorical results.

In general, the number of CT scans per patient for those individuals from the 2^nd^ to the 5^th^ quintile decreased by a factor of 0.985 to 0.973 (1 to 3%) when compared to the reference category (1^st^ SES quintile or less vulnerable SES), (Table 2). It was statistically significant for the 4^th^ quintile only. The overall effect was driven by the biggest ACs (Catalonia and Madrid community).

**Table 2:** Incidence rate ratio (IRR) estimates and 95% confidence intervals (95% CI) for the association between number of CT scans per patient and socioeconomic status measured by the Urban Vulnerability Synthetic Index (UVSI), controlling for sex and age at the time of the last CT scan, as well as for Autonomous Community.

Model*	IRR	95% CI	p-value
**Number of CT scans, UVSI (continuous)**	0.944	(0.829–1.075)	0.387
Number of CT scans, UVSI*			
1 SES quintile (less vulnerable)—Ref. category	1.000		
2 SES quintile	0.985	(0.958–1.013)	0.287
3 SES quintile	0.983	(0.950–1.016)	0.308
4 SES quintile	0.973	(0.948–0.999)	0.043
5 SES quintile (more vulnerable)	0.987	(0.935–1.042)	0.637
**Number of CT scans, UVSI in Catalonia**			
1 SES quintile (less vulnerable)—Ref. category	1.000		
2 SES quintile	0.976	(0.951–1.001)	0.062
3 SES quintile	0.980	(0.953–1.007)	0.146
4 SES quintile	0.955	(0.929–0.981)	0.001
5 SES quintile (more vulnerable)	0.957	(0.929–0.985)	0.003
**Number of CT scans, UVSI in Valencia Community**			
1 SES quintile (less vulnerable)—Ref. category	1.000		
2 SES quintile	0.936	(0.881–0.994)	0.031
3 SES quintile	0.924	(0.870–0.981)	0.009
4 SES quintile	0.990	(0.935–1.049)	0.745
5 SES quintile (more vulnerable)	1.068	(1.007–1.133)	0.030
**Number of CT scans, UVSI in Murcia Region**			
1 SES quintile (less vulnerable)—Ref. category	1.000		
2 SES quintile	0.960	(0.830–1.111)	0.585
3 SES quintile	0.907	(0.777–1.060)	0.221
4 SES quintile	0.958	(0.843–1.088)	0.505
5 SES quintile (more vulnerable)	0.964	(0.856–1.086)	0.548
**Number of CT scans, UVSI in Navarre**			
1 SES quintile (less vulnerable)—Ref. category	1.000		
2 SES quintile	1.029	(0.984–1.077)	0.209
3 SES quintile	1.021	(0.970–1.075)	0.421
4 SES quintile	1.016	(0.957–1.079)	0.600
5 SES quintile (more vulnerable)	0.963	(0.877–1.057)	0.425
**Number of CT scans, UVSI in Madrid Community**			
1 SES quintile (less vulnerable)—Ref. category	1.000		
2 SES quintile	1.034	(0.990–1.081)	0.135
3 SES quintile	1.030	(0.989–1.073)	0.159
4 SES quintile	0.974	(0.940–1.010)	0.150
5 SES quintile (more vulnerable)	0.961	(0.928–0.996)	0.029

* including Autonomous community of residence as a random effect

The effect differed by AC of residence. In Catalonia, subjects with lower SES showed a decrease in CT scans per patient (statistically significant for the 4^th^ and 5^th^ quintiles) compared with the most affluent quintile. In Valencia community, compared to the reference category, subjects from the 2^nd^, 3^rd^ and ^4th^ socioeconomic quintile had a lower rate of CT scans per patient (statistically significant for the 2^nd^ and 3^rd^ quintile), and those belonging to the most deprived socioeconomic level (5^th^ quintile) showed an increased rate (IRR = 1.068; 95% CI = 1.007–1.133) (Table 2). In Madrid community, the rate in the 2^nd^ and 3^rd^ quintiles increased by a factor of 1.03; and it decreased in the most deprived socioeconomic quintiles compared to the reference category (1^st^ quintile; less deprived). In Navarra and Murcia region, though there was variability in the IRR, numbers were small and there was no statistical evidence for a difference across SES levels.

**Fig 3:**
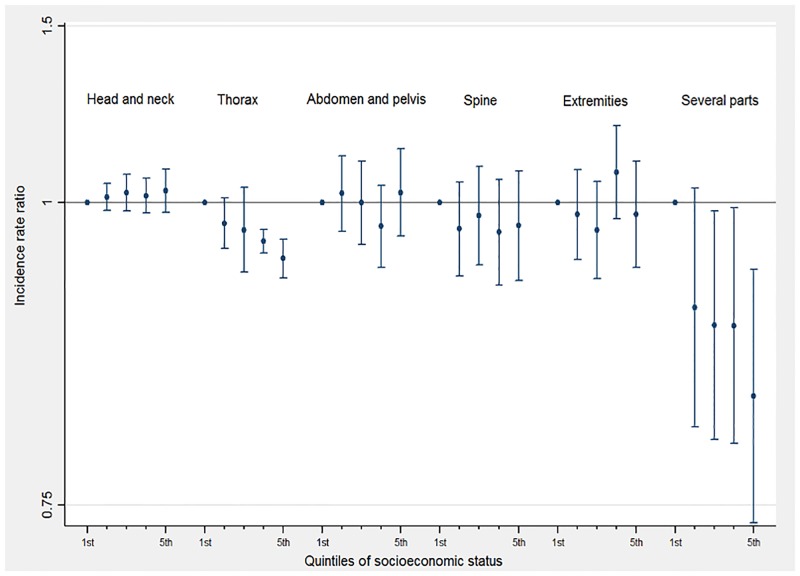
CT scan multiplying factor (CT scan incidence rate ratio) by SES quintile for different body parts scanned in children and young adults (0–20 years old).

No major differences were observed in rates of CT scans per patient by UVSI for most body parts scanned. For “thorax” examinations, the rate per patient decreased with decreasing SES when compared with the more privileged group (1^st^ quintile). For “several parts” examinations, the rate decreased by a factor of 0.786 to 0.641 for those patients belonging to lower socioeconomic groups (quintiles 2^nd^ to 5^th^) compared to the reference category (Fig 3).

### Comparison of individual SES indicators with the UVSI

The supplementary figure (S1 Fig) shows a strong correlation (correlation coefficient 0.82–0.86) between UVSI and “temporary employment”, “unskilled work” and “illiteracy”. Table 3 shows the relationship between the number of CT scans per patient and each of the socioeconomic indicators composing the UVSI.

**Table 3:** Rate of CT scans per patient by socioeconomic status quintile using the individual socioeconomic indicators that constitute the UVSI as the SES measures.

Rate in the number of CT scans per patient	CT scans performed from 1991 to 2005 (N = 37,096 subjects)				CT scans performed from 2006 to 2013 (N = 86,633 subjects)							
	CENSUS 2001				CENSUS 2001				CENSUS 2011			
	% Categories in the quintiles of the indicator	IRR	95% CI	p-value	% Categories in the quintiles of the indicator	IRR	95% CI	p-value	% Categories in the quintiles of the indicator	IRR	95% CI	p-value
By SES quintiles based on % of unemployment in the census tract												
SES 1	(0.0–8.2)	1	-	-	(0.0–8.2)	1	-	-	(1.9–17.6)	1	-	-
SES 2	(8.2–10.1)	0.968	(0.949–0.988)	0.002	(8.2–10.1)	0.970	(0.944–0.996)	0.026	(17.6–22.8)	1.006	(1.001–1.011)	0.016
SES 3	(10.1–11.8)	0.971	(0.954–0.988)	0.001	(10.1–11.8)	0.952	(0.905–1.002)	0.058	(22.8–28.1)	1.005	(0.993–1.017)	0.438
SES 4	(11.8–14.1)	0.965	(0.913–1.020)	0.209	(11.8–14.1)	0.949	(0.902–0.998)	0.043	(28.1–35.2)	1.002	(0.986–1.018)	0.800
SES 5	(14.1–39.1)	0.960	(0.903–1.022)	0.200	(14.1–39.1)	0.937	(0.892–0.984)	0.009	(35.2–93.9)	1.005	(0.959–1.052)	0.844
By SES quintiles based on % of unemployment in young people in the census tract												
SES 1	(0.0–10.9)	1	-	-	(0.0–10.9)	1	-	-	(1.8–25.2)	1	-	-
SES 2	(10.9–13.5)	0.985	(0.960–1.012)	0.280	(10.9–13.5)	0.968	(0.937–0.999)	0.045	(25.2–35.8)	0.998	(0.963–1.036)	0.931
SES 3	(13.5–15.8)	0.962	(0.942–0.981)	0.000	(13.5–15.8)	0.960	(0.931–0.990)	0.009	(35.8–46.5)	1.033	(0.992–1.075)	0.115
SES 4	(15.8–18.9)	0.955	(0.899–1.015)	0.138	(15.8–18.9)	0.952	(0.897–1.010)	0.101	(46.5–60.4)	1.026	(1.010–1.041)	0.001
SES 5	(18.9–54.0)	0.919	(0.888–0.952)	0.000	(18.9–54.0)	0.946	(0.883–1.014)	0.116	(60.4–100.0)	0.993	(0.972–1.014)	0.503
By SES quintiles based on % of temporary work in the census tract												
SES 1	(3.9–16.8)	1	-	-	(3.9–16.8)	1	-	-	(0.8–10.3)	1	-	-
SES 2	(16.8–20.3)	0.980	(0.967–0.992)	0.001	(16.8–20.3)	0.957	(0.938–0.976)	0.000	(10.3–14.1)	0.994	(0.971–1.017)	0.586
SES 3	(20.3–23.9)	0.982	(0.968–0.996)	0.010	(20.3–23.9)	0.956	(0.934–0.978)	0.000	(14.1–18.0)	1.001	(0.970–1.032)	0.955
SES 4	(23.9–28.6)	0.986	(0.964–1.009)	0.240	(23.9–28.6)	0.963	(0.936–0.991)	0.010	(18.0–23.7)	0.988	(0.944–1.035)	0.611
SES 5	(28.6–81.8)	1.000	(0.900–1.112)	0.999	(28.6–81.8)	0.979	(0.912–1.050)	0.543	(23.7–100.0)	1.005	(0.947–1.067)	0.858
By SES quintiles based on % of unskilled work in the census tract												
SES 1	(0.0–6.6)	1	-	-	(0.0–6.6)	1	-	-	(0.3–4.8)	1	-	-
SES 2	(6.6–9.0)	0.978	(0.928–1.031)	0.407	(6.6–9.0)	1.013	(0.993–1.034)	0.203	(4.8–8.2)	1.003	(0.982–1.024)	0.807
SES 3	(9.0–11.7)	0.972	(0.922–1.024)	0.283	(9.0–11.7)	1.008	(0.974–1.044)	0.649	(8.2–12.0)	1.024	(1.009–1.040)	0.002
SES 4	(11.7–15.0)	1.003	(0.976–1.030)	0.845	(11.7–15.0)	0.982	(0.967–0.996)	0.015	(12.0–17.5)	1.013	(0.976–1.052)	0.483
SES 5	(15.0–77.1)	0.963	(0.906–1.023)	0.221	(15.0–77.1)	0.973	(0.935–1.013)	0.181	(17.5–87.5)	0.984	(0.940–1.030)	0.487
By SES quintiles based on % of illiterate population in the census tract												
SES 1	(0.0–6.3)	1	-	-	(0.0–6.3)	1	-	-	(0.2–4.2)	1	-	-
SES 2	(6.3–9.9)	1.029	(0.948–1.118)	0.493	(6.3–9.9)	1.028	(1.002–1.055)	0.032	(4.2–7.2)	1.003	(0.993–1.013)	0.568
SES 3	(9.9–14.0)	1.045	(0.948–1.151)	0.381	(9.9–14.0)	0.998	(0.975–1.022)	0.888	(7.2–10.5)	1.001	(0.984–1.018)	0.911
SES 4	(14.0–20.0)	1.059	(0.945–1.186)	0.328	(14.0–20.0)	0.992	(0.960–1.025)	0.617	(10.5–15.4)	0.996	(0.961–1.032)	0.805
SES 5	(20.0–77.7)	1.045	(0.927–1.178)	0.469	(20.0–77.7)	0.989	(0.944–1.037)	0.654	(15.4–66.8)	0.978	(0.932–1.026)	0.357

Comparison of patients with CT scans performed between 1991 and 2005 with those with CT scans performed between 2006 and 2013. In the later group, results based on both the 2001 and 2011 censuses are shown for comparison. All estimations used the distributional quintile cut-off points, that is, groups comprising 20% of the population aged 0 to 20, and are adjusted by sex and age at the time of the last CT scan and include Autonomous Community of residence as a random factor.

### Comparison using up-to-date SES indicators

For those who received a CT scan from 2006 onwards, results are shown using both 2001 and 2011 census data. With the 2011 census data, an increased IRR was seen for subjects in the 2^nd^, 3^rd^, 4^th^ and 5^th^ quintiles of “unemployment” (statistically significant for the 2^nd^ quintile). When using “youth unemployment” as the SES indicator, an increased IRR was observed for the 3^rd^ and 4^th^ quintiles, and a decrease for the 2^nd^ and 5^th^, compared to the 1^st^ (less vulnerable). No major differences were observed using indicators of “temporary work” and “illiteracy”. When using cut-off points based on quintiles of the 2001 indicators, one notes that “unemployment” and “youth unemployment” has grown considerably over the 2001–2011 time period, while “illiteracy” has declined.

Using indicators based on the 2001 census in this population, unlike the 2011 indicators, reduced IRRs were observed in the 2^nd^, 3^rd^, 4^th^ and 5^th^ quintiles of global “unemployment”, ”youth unemployment” and “temporary work”. When using “illiteracy” as the SES indicator, the rate of CT scans per person decreased by a factor of 0.989 to 0.998 depending on the quintile, and increased by a factor of 1.028 for those from the 2^nd^ SES quintile. Finally, when the 2001 census data was used to assign a SES to those whose last registered CT scan happened prior to 2006, a decrease in IRR for subjects in the 2^nd^ to 5^th^ quintiles of the indicators of “unemployment”, “youth unemployment” and “temporary work” was observed in the population similar to decreases seen in those with later scans using the 2001 census. No association was seen with the indicators for “unskilled work” and “illiteracy”.

Compared to the other indicators, the “percentage of unemployment among young people (16 to 29 years old)” was the socioeconomic dimension that showed the greatest changes between the 2001 and 2011 census, since the SES quintile remained unchanged for only 21.7% of all geocoded participants over the 10 years between both censuses. The second most unstable socioeconomic indicator was the “percentage of unemployment among active population (16 to 65 years old)” with 30.4% of the subjects SES unchanged. This was followed by “percentage of temporary employment”, “percentage of unskilled employment” and the “percentage of illiterate population” with 35.1%, 36.5% and 39.6% of the subjects SES unchanged, respectively (data not shown).

## Discussion

Using data from the Spanish EPI-CT subcohort and the 2001 census, we found evidence of socio-economic variation in a group of CT scanned young individuals, mainly related to AC of residence—with a higher proportion of scanned patients in the most affluent groups in Catalonia and Navarra, and the opposite in the Madrid, Murcia and Valencia communities. Although social security coverage in Spain is universal, it is administered at the autonomic level since 2001. While there was little difference by SES for most types of scans, we noted a higher rate of scans of multiple body parts in the less affluent categories, possibly reflecting a higher rate of accidents and injuries.

A more in-depth analysis, controlling by age and sex of the patient, showed that although the rate of CT scans per person was slightly lower in the most disadvantaged groups than in the less vulnerable SES group, the difference did not reach statistical significance. This suggests that overall, when all ACs are combined, there are no SES differences in the chance to receive a CT scan for the diagnosis and follow-up of medical conditions. The adjusted relationship between the CT scan rate per person and SES was U-shaped, with the most disadvantaged SES group having a higher CT scan rate per person than the 2^nd^, 3^rd^ and 4^rth^ SES quintiles. When the relationship between SES and CT scan rate per patient was studied by AC, however, differences were observed: in Catalonia a decreasing rate of CT scans per patient was observed with decreasing SES, while in Madrid community the decrease was only in the lowest socioeconomic groups. These observed disparities by Autonomous Community are likely to be related to the territorial differences in health care management and the ensuing effects on access and use of health care facilities. They may also be related to clinical practice, and to the technological supply of scanners available in each community. The widest differences in the rate of CT scans per person by SES were observed in the Valencia and Madrid Communities.

It is worth noticing that the SES distribution of scanned individuals in our cohort differed from that in the general Spanish population: there was an overrepresentation of the more advantaged population in Catalonia, Valencia Community and Navarre, and an overrepresentation of disadvantaged population in Murcia and Madrid Community [1].

Interestingly, when the relative socioeconomic position of the patients was measured using the 2001 five UVSI constituent indicators, a significant decrease in the number of CT scans per patient was seen with decreasing SES, both for the indicators related to “unemployment” and “temporary employment”. Including a more up-to-date (2011 census data) SES information led to results in different directions when “unemployment” and “temporary employment” were used as SES indicators.

The study pointed out that “unemployment” and “youth unemployment” were the social dimensions most impacted by the global financial crisis that started in 2007–08. Job insecurity and unemployment are considered stressors related to poor health [21,22], although the causal direction of these relationship is not completely evident. When using data prior to 2006, the results suggested that the rate of CT scans per person decreased with increasing unemployment and job insecurity in the children’s area of residence. With more recent data, the results specifically for “unemployment” suggested the opposite, reflecting a potential higher injury and disease rate in the most deprived areas.

This study also outlined a socioeconomic mobility among quintiles for more than 2 thirds of the studied population between 2001 and 2011 and, therefore, it highlighted the potential relevance of having more than one-point-in-time measure of SES in longitudinal studies. Major societal changes, such as the 2007/2008 financial crisis, may have a profound effect on class relations, reshaping socioeconomic class ties over time [15].

When analysing the impact of each SES dimension on the number of CT scans per person we need to bear in mind that composite measures and individual indicators may capture different aspects of societal relationships. Therefore, it is not unusual to find that some individual measures are weakly correlated with the SES composite index (UVSI) and that the observed relation with CT scanning varies with the indicator chosen. Employment and working conditions-based SES measures may be strongly related to social status and privilege and therefore, in our study they may be capturing easier access and better quality health care [23]. The education-based indicator may reflect the health-related knowledge asset of the progenitors [23], and may measure the parents’ choices and constraints over the health of the progeny. Thus, the individual indicators of SES may not be equally significant in their impact on health care access and use, and as a consequence, may not contribute equally to the cumulative rate of CT scans per patient. This is important to consider when comparing results of studies which may have used different socioeconomic indicators.

The findings in our study differed from those in the European and North American studies reporting the use of CT scan by SES, where a highest CT scan use was observed in vulnerable areas compared to the CT scan use in less deprived areas [6–9]. Head scans were significantly associated to deprivation in the UK study [6], suggesting that head injuries were the main reason behind the cumulative excess radiation doses received by the study population. In none of the studies though, CT scan indication data was available to help deciphering the observed results, although in the paper by Miller [7], information on concurrent comorbidity of the patient was available and associated to cumulative radiation exposure from diagnostic imaging. Interestingly enough, these results were seen in countries with different health care systems (public vs. private). On the other hand, an Australian study reported the opposite relationship [4], where people living in more deprived areas were slightly less exposed to the radiation delivered in a CT scan than in the less vulnerable areas. As mentioned before, it is difficult to compare our findings with those observed in previous studies, given the fact that they may result from the use of different SES indicators. For example, the Dutch study used measures of material deprivation (home income and home value) whereas the Townsend deprivation index used in UK incorporates employment in addition to material deprivation indicators [6,8]. In the Australian [4], German [9] and in the present study the indexes used to explain the socioeconomic reality of the study population were more relatable, although the overlapping of the social dimensions included in each index was incomplete.

The strengths of this analysis include its large young population with geographically-rich socioeconomic information, the use of wide-range univariated SES indicators, and the inclusion of an updated SES measure. There are also, however, several limitations that should be acknowledged. The 5 socioeconomic indicators used in these analyses were based on the 2001 and 2011 SES census household surveys which presented some differences. Specifically, the former was a non exhaustive sampling of approximately 12% of all Spanish households. Additionally, the 2011 census had no information on the 5 indicators of interest for 1 to 9% of the census tracts. There is a small but non-negligible possibility that the non exhaustive sampling of 2011 census data could have affected the precision and accuracy of our categorisation. Additionally, when using the 2001 census data in subjects for whom the hospitals had no updated address after 2006, we were assuming that they had not changed addresses, so there is some potential for socioeconomic status misclassification.

## Conclusions

Overall we observed some differences in the SES distribution of scanned patients by autonomous community. At the national level there were, however, no major differences in the frequency of CT scans per patient by SES in public and autonomically-subsidised hospitals by our main measure of SES. This finding may be useful for epidemiological studies, where the association between a health outcome and CT scan radiation is usually thought to be confounded by SES. Given the results of this study, SES can be ruled out as a confounder because it had no impact on the number of CT scans per patient (cumulative radiation). The use of different indicators and of data on SES collected in different time points led to different relations between SES and frequency of CT scans, outlining the difficulty of adequately capturing the social and economic dimensions which may affect health and health service use.

## Supporting information

S1 Fig Graphical correlation and Spearman coefficients between the Urban Vulnerability Synthetic Index and the univariate indicators that compound the Index, standardized to the national level of each indicator. The x-axis scales vary accordingly to the number of times the percentage (%) of, for example, unemployed population aged 16–29 years out of the total active population aged 16 to 29 in a census-tract is above or below the national value of unemployed population in this age range.
(TIF)

S1 Table Distribution of demographic characteristics in children and young adults 0–20 years by geocoding success. (DOCX)
