# Supplementary figures and images for: CT scan exposure in Spanish children and young adults by socioeconomic status: Cross-sectional analysis of cohort data
Source: PLoS One. 2018 May 3;13(5):e0196449. doi: 10.1371/journal.pone.0196449 (PMC5933709; doi:10.1371/journal.pone.0196449)

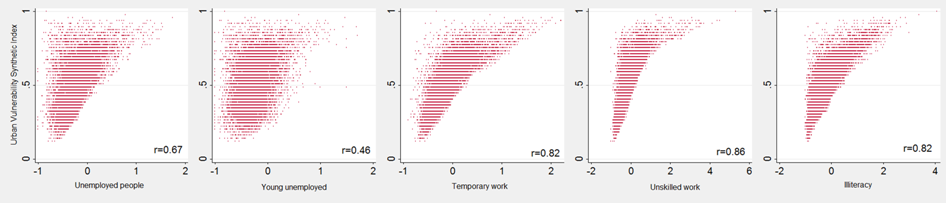

Supplement: S1 Fig — The x-axis scales vary accordingly to the number of times the percentage (%) of, for example, unemployed population aged 16–29 years out of the total active population aged 16 to 29 in a census-tract is above or below the national value of unemployed population in this age range. (TIF) [file pone.0196449.s001.tif]
