# Supplementary material for: CT scan exposure in Spanish children and young adults by socioeconomic status: Cross-sectional analysis of cohort data
Source: PLoS One. 2018 May 3;13(5):e0196449. doi: 10.1371/journal.pone.0196449 (PMC5933709; doi:10.1371/journal.pone.0196449)
Supplement: S1 Table — (DOCX) [file pone.0196449.s002.docx]

**S1 Table. Distribution of demographic characteristics in children 0-20 years by geocoding success**

|  |  | **No geocoded subjects** | **Geocoded subjects** |
| --- | --- | --- | --- |
|  |  | N = 31,441 | N = 123,729 |
| **Sex** | Male | 18,065 (20.6%) | 69,680 (79.4%) |
|  | Female | 13,515 (20.0%) | 54,049 (80.0%) |
| **Autonomous Community where they had their first CT scan** | Catalunya | 13,397 (18.0%) | 60,853 (82.0%) |
|  | Valencia Community | 8,105 (30.7%) | 18,289 (69.3%) |
|  | Murcia region | 892 (16.6%) | 4,482 (83.4%) |
|  | Navarre | 697 (7.1%) | 9,184 (93.0%) |
|  | Madrid Community | 8,489 (21.5%) | 30,921 (78.5%) |
| **Year of birth** | 1970 - 1975 | 206 (25.0%) | 617 (75.0%) |
|  | 1976 - 1980 | 592 (17.0%) | 2,894 (83.0%) |
|  | 1981 - 1985 | 1,638 (14.5%) | 9,644 (85.5%) |
|  | 1986 - 1990 | 5,262 (20.1%) | 20,952 (79.9%) |
|  | 1991 - 1995 | 7,854 (21.5%) | 28,766 (78.6%) |
|  | 1996 - 2000 | 6,102 (20.8%) | 23,247 (79.2%) |
|  | 2001 - 2005 | 4,788 (19.5%) | 19,800 (80.5%) |
|  | 2006 - 2010 | 4,050 (21.8%) | 14,530 (78.2%) |
|  | 2011 - 2013 | 1,088 (24.9%) | 3,279 (75.1%) |
| **Number of CT scans per person** | 1 | 22,831 (20.3%) | 89,709 (79.7%) |
|  | 2 | 7,616 (20.3%) | 29,940 (79.7%) |
|  | 3 - 10 | 829 (21.4%) | 3,053 (78.7%) |
|  | 11 -20 | 269 (22.9%) | 905 (77.1%) |
|  | > 20 | 35 (22.3%) | 122 (77.7%) |
